# Pork as Food

**Published:** 1886-10

**Authors:** 


### *PORK AS FOOD.*

### The Two Parasitic Evils Developed in the Tissues of Hogs.

The prejudice against the flesh of swine as human food is as old as history. If it has any foundation in nature besides the filthy manner in which the hog is generally kept, it is because the hog is more subject to disease, or at least to a certain class of diseases, than other domestic animals. Its omniverous appetite makes it liable to certain diseases from which the exclusively vegetable-eating animals are comparatively, if not entirely, exempt.

The two evils most complained of are tape-worm and trichina—both parasitic. Both of these are developed in the animal tissues, and it is very doubtful if they ever afflict animals that do not eat animal food, or in some way get animal products into their stomachs and intestinal canals.

The tape-worm, when encysted in the tissues of the hog, has the name of measles—though wholly unlike measles in the human species—the pork containing these encysted worms is known as "measly pork." When taken into the human stomach, they are liable to develop there in the form of the loathsome creature known as tape-worm. This is not necessarily fatal, but very annoying. In modern times, it is successfully removed by the skillful physician without pain or injury to the patient.

Trichina is a parasite much more to be dreaded even than the tape-worm. It is liable to infect the human system in such numbers as not only to be very painful, but fatal, and we believe there is no known remedy. It is encysted in the flesh of the animal, in a dormant state, like the tape-worm. When the trichina enters the human stomach, it attaches itself to the mucous membrane and there awakens to all the activity of breeding thousands, if not millions, of its kind. These young trichina at once start out to find a place in which to encyst themselves for a dormant rest and await a resurrection by having the flesh in which they are imbedded eaten by some animal, brute or human, when they will repeat the roll of their progenitors.

It is when passing from the stomach and intestines to the muscles that the trichina give such pain, and frequently cause death. Pushing their way through the tissues, they cause great irritation and inflammation, resulting in death when the effects become unendurable. While in this active state of migration, seeking a home, they are as liable to be found in one class of tissues as in another. Hence, lard that has not been exposed to a heat of at least 212 degrees Fahrenheit, is just as likely to contain them, if the animal was killed when the trichina were in a state of activity, as in any other part or product of the hog.

The only way to avoid the evil and suffering from tape-worms and trichina is to either wholly abstain from eating pork, or to be sure that it is thoroughly cooked so as to distroy the vitality of the encysted parasite. The muscle of the hog makes exceedingly palatable food, and many enjoy eating the fat. In spite of all the terrors and drawbacks, pork is a common and popular article of food. So long as animal flesh continues to be eaten by man, we presume pork will be eaten. As there is no cure for one of the diseases imparted by it, the safety of the public health demands that hogs be kept in a cleanly manner, fed only vegetable food and such animal products as belong to the dairy, and that the meat—of all kinds, as for that matter—shall be sufficiently cooked to destroy all animal life. Nothing less than subjecting every particle of it to a heat of at least 212 degrees will do it.—*National Live-Stock Journal.*

